# Association between Clinical and Laboratory Markers and 5-year Mortality among Patients with Stroke

**DOI:** 10.1038/s41598-019-47975-y

**Published:** 2019-08-08

**Authors:** Chien-Min Chen, Meng Lee, Yao-Hsu Yang, Shih-Shin Huang, Chu-Hsu Lin

**Affiliations:** 1 Department of Physical Medicine and Rehabilitation, Chang Gung Memorial Hospital, Chiayi, Taiwan; 2 School of Medicine, College of Medicine, Chang Gung University, Taoyuan, Taiwan; 3 Department of Neurology, Chang Gung Memorial Hospital, Chiayi, Taiwan; 4 Department of Traditional Chinese Medicine, Chang Gung Memorial Hospital, Chiayi, Taiwan; 5 School of Traditional Chinese Medicine, College of Medicine, Chang Gung University, Taoyuan, Taiwan; 6 Health Information and Epidemiology Laboratory of Chang Gung Memorial Hospital, Chiayi, Taiwan

**Keywords:** Risk factors, Stroke

## Abstract

Factors influencing long-term stroke mortality have not been comprehensively investigated. This study aimed to identify the baseline clinical, laboratory, demographic/socioeconomic, and hospital factors influencing 5-year mortality in patients with first stroke. Total 3,956 patients with first-stroke hospitalization from 2004 to 2008 were connected to the longitudinal National Health Insurance Research Database. Post-admission baseline data that significantly increased 5-year mortality were red cell distribution width (RDW) >0.145 (adjusted hazard ratio [aHR] = 1.71), hemoglobin <120 g/L (aHR = 1.25), blood sugar <3.89 mmol/L (70 mg/dL)(aHR = 2.57), serum creatinine >112.27 μmol/L (aHR = 1.76), serum sodium <134 mmol/L (aHR = 1.73), body mass index (BMI) < 18.5 kg/m^2^ (aHR = 1.33), Glasgow Coma Scale <15 (aHR = 1.43), Stroke Severity Index ≥20 (aHR = 3.92), Charlson–Deyo Comorbidity Index ≥3 (aHR = 4.21), no rehabilitation (aHR = 1.86), and age ≥65 years (aHR = 2.25). Hemoglobin, RDW, blood sugar, serum creatinine and sodium, BMI, consciousness, stroke severity, comorbidity, rehabilitation, and age were associated with 5-year mortality in patients with first stroke.

## Introduction

In 2017, stroke was found to be the second leading cause of mortality worldwide^1^ and the fourth leading cause of mortality in Taiwan^2^. Among 291 diseases and injuries worldwide, stroke was found to be the third leading cause of burden, measured by disability-adjusted life years^3^.

Some factors, such as age^4^, sex^4,5^, stroke type^4^, stroke severity^4^, Charlson–Deyo Comorbidity Index (CCI)^4^, rehabilitation^4,6^, and income^7^, have been identified in few large-scale studies^4–7^ using national databases as predictors of long-term mortality in patients with stroke. Nationwide studies included a large number of patients; however, these studies did not include overall baseline markers, particularly clinical and laboratory data, for further analysis.

Reviewing past literature, regardless of the post-stroke duration, some factors are known to affect the mortality in patients with stroke. Red blood cell count (RCC)^8^, mean corpuscular volume (MCV)^8^, mean platelet volume^9^, low diastolic blood pressure^10^ during admission, pyrexia^10^, and severe obesity^11^ were factors associated with post-stroke mortality in patients with ischemic stroke. In addition, anemia^12^ and hospital size^13^ influence post-stroke mortality in patients with hemorrhagic stroke. Moreover, white blood cell count^14^, serum creatinine levels^14^, hyperglycemia^15^, hyponatremia^16^, red cell distribution width (RDW)^17^, systolic blood pressure^14^, hyperthermia^18^, and residential environment^19^ influence post-stroke mortality in patients with combined ischemic and hemorrhagic stroke.

To our best knowledge, no large-scale study has comprehensively addressed the factors associated with long-term mortality in patients with first stroke, including clinical data, laboratory data, demographic/socioeconomic status, and hospital accreditation level. Among these collectable baseline markers, some have greater impact on long-term mortality in patients with first stroke. We connected hospital research database and the National Health Insurance Research Database (NHIRD) in Taiwan to determine the association between baseline data and long-term mortality in patients with first stroke.

## Methods

### Source of data

The Chang Gung Research Database (CGRD), sourced from the 3600-bed Linko Chang Gung Memorial Hospital (CGMH) medical center, the 2600-bed Kaohsiung CGMH medical center, the 1300-bed Chiayi CGMH regional hospital, and the 750-bed Keelung CGMH regional hospital contained medical information such as de-identified personal data, including data from the research database, laboratory, nursing, and claims systems of admissions and emergency departments. Medical information of patients with stroke from the four hospitals between 2004 and 2008 were retrieved from CGRD, according to the first diagnosis code at discharge from the International Classification of Diseases, 9^th^ edition, Clinical Modification (ICD-9-CM codes 430–434).

NHIRD included de-identified personal data released by the National Health Research Institutes for public research purposes. Longitudinal medical information of all patients with stroke according to the first diagnosis code at discharge (ICD-9-CM codes 430–434) from 1997 to 2013 was obtained from NHIRD, including registry for beneficiaries, registry for contracted medical facilities, and order details of claims for inpatient, outpatient, and emergency departments. The Institutional Review Board for Human Studies of the Chang Gung Memorial Hospital approved the study protocol (approval number: 201700742B0C603) and waived the requirement of informed consent.

### Database connection for study patients

Data of the patients with inpatient claims including primary diagnosis of stroke at discharge (ICD-9-CM codes 430–434) were separately collected from CGRD and NHIRD between 2004 and 2008. Subarachnoid and intracerebral hemorrhage, categorized as ICD-9-CM 430 and 431–432, were combined as hemorrhagic stroke. Cerebral infarction, categorized as 433–434, was represented as ischemic stroke.

Patients were excluded according to the following criteria: (1) patients with ICD-9-CM codes 430–434, 436–437 (ill-defined, cerebrovascular disease), and 438 (late effects of cerebrovascular disease) for outpatient, emergency department, and inpatient claims of NHIRD from 1997 to 2003, respectively, because they were not considered to be first stroke patients; (2) those with no definite date of discharge for first-stroke hospitalization; and (3) those withdrew from National Health Insurance within 5 years after the index date.

**Figure 1:**
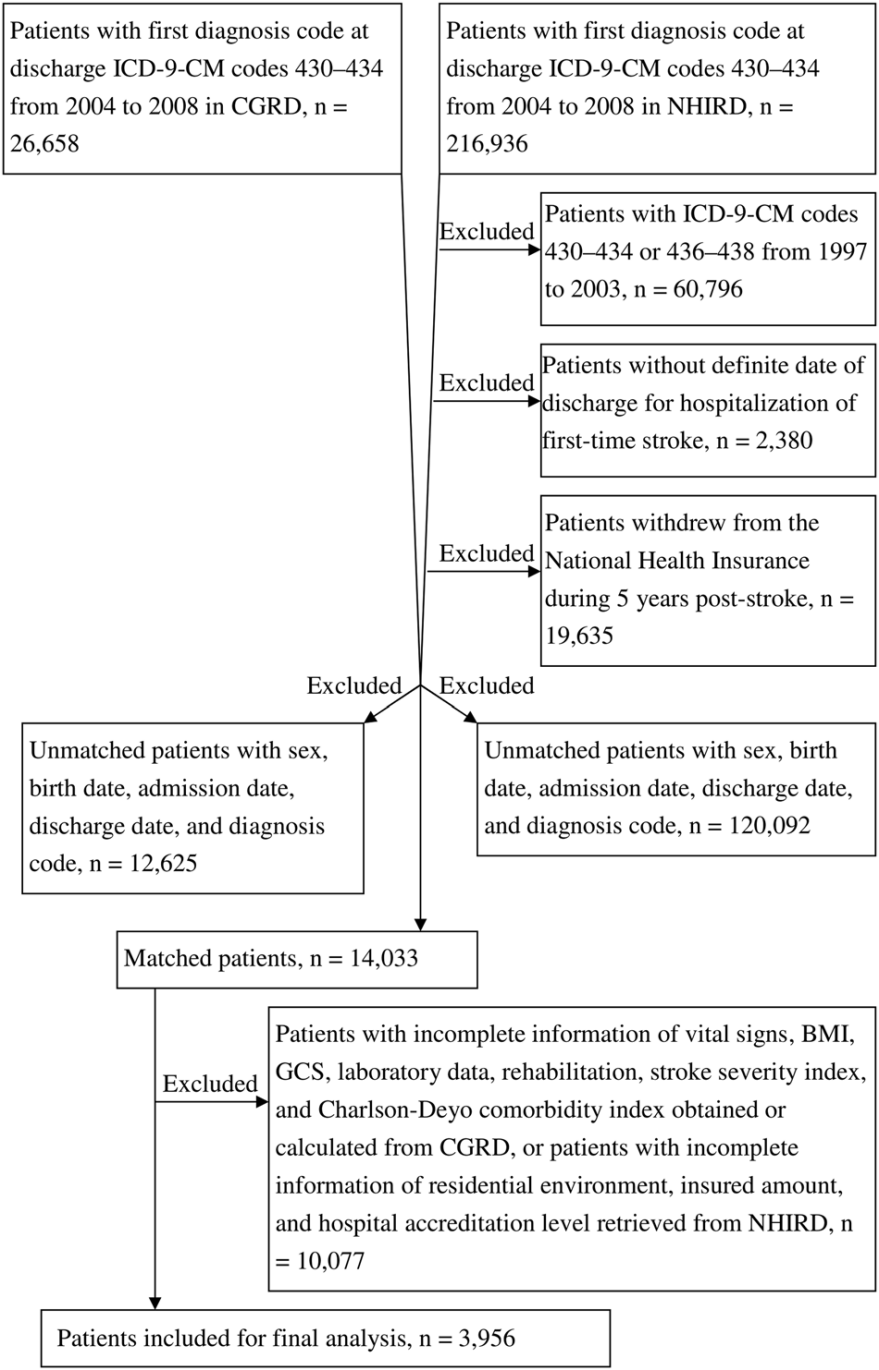
Flowchart of study patient selection.

To identify CGRD patients with stroke in NHIRD, we connected CGRD with NHIRD by matching five non-unique characteristics (sex, birth date, admission date, discharge date, and five main diagnoses) of patients with primary stroke diagnoses from 2004 to 2008. If a registry case was connected to more than one patient in NHIRD, the case was excluded from the study. Furthermore, matched patients with incomplete data regarding potential factors were excluded. Finally, the remaining 3,956 patients comprised the study cohort (Fig. 1).

### Collection of potential factors

Clinical data, including baseline data after hospital admission (at the emergency department or ward, whichever appeared first) of body mass index (BMI; <18.5, 18.5–24.9, and ≥25 kg/m^2^), Glasgow Coma Scale (GCS; 15 and <15), body temperature (<35.7, 35.7–37.3, and >37.3 °C), systolic blood pressure (<120, 120–139, and ≥140 mmHg), and diastolic blood pressure (<80, 80–89, and ≥90 mmHg), were obtained from CGRD. The reference range for BMI from the World Health Organization^20^ is 18.5–24.9 kg/m^2^; thus, we categorized patients into BMI groups of <18.5, 18.5–24.9, and ≥25 kg/m^2^. Any GCS score of <15 was considered as impaired consciousness^21^; thus, we categorized the GCS score into 15 and <15. The reference range for temperature and blood pressure was according to studies performed by Obermeyer *et al*.^22^ and Whelton *et al*.^23^, respectively. The Stroke Severity Index (SSI)^24^ and the CCI^25,26^ scores were measured, and information regarding inpatient rehabilitation during first-stroke hospitalization was obtained from the claims of CGRD. The stroke type (ischemic or hemorrhagic) was obtained from CGRD or NHIRD based on the primary diagnosis. Hospital admission was defined as a patient who entered the hospital, through the emergency department or ward, for inpatient service.

The SSI score, including several clinical procedures and factors regarding first-stroke hospitalization data, was used to examine stroke severity. The CCI score, calculated using weighted-summary measures of important concomitant diseases selected from the ICD-9-CM codes at discharge, was used to examine the complexity of comorbidity. ICD-9-CM codes associated with cerebrovascular disease and hemiplegia were not used for scoring CCI.

First laboratory data after admission, including blood sugar (<3.89, 3.89–7.77, and >7.77 mmol/L), hemoglobin (<120, 120–175, and >175 g/L), MCV (<80, 80–100, and >100 fL), RDW (<0.115, 0.115–0.145, and >0.145), RCC (<4.0, 4.0–5.9, and >5.9 × 10^12^/L), white blood cell count (<3.5, 3.5–11.0, and >11.0 10^9^/L), serum creatinine (<38.90, 38.90–112.27, and >112.27 μmol/L), serum sodium (<134, 134–148, and >148 mmol/L), and serum potassium (<3.6, 3.6–5.0, and >5.0 mmol/L), were obtained from the laboratory data system of CGRD. Since reference ranges of laboratory values may vary among different sources, we used the reference ranges supplied by the Department of Laboratory Medicine of Chang Gung Memorial Hospital, recorded on June 8, 2018. If the reference range of one laboratory value was different between men and women, the upper limit of the interval for stratifying the laboratory value was set as the greater value in the upper limit of the reference range of men or women; similarly, the lower limit of the interval for stratifying the laboratory value was set as the lesser value in the lower limit of the reference range of men and women.

Demographic/socioeconomic status, including age at stroke onset (<45, 45–64, and ≥65) and sex (men or women), was obtained using either CGRD or NHIRD. Data regarding insured amount per month (<United States dollar [USD] 700 and ≥USD 700) and residential environment (seven urbanization levels)^27^ were obtained from NHIRD. Residential environment was categorized as urban, including urbanization levels 1 and 2; suburban, including urbanization levels 3 and 4; and rural, including urbanization levels 5, 6, and 7. The hospital factor, hospital accreditation level (medical center and regional hospital), was obtained from NHIRD. The threshold of USD 700 as designated by the Bureau of National Health Insurance, was equivalent to the lowest insured monthly salary (New Taiwan dollar [NTD] 21000; NTD:USD = 30:1) for Taiwanese employees during the years of data collection.

### Definition of endpoint and mortality

The information regarding follow-up and all-cause mortality of the study patients was sourced from NHIRD. The index date was defined as the date of hospital admission of the patient with first stroke. All study patients were examined from the index date to the endpoint, which was defined as the date of all-cause mortality or 5 years after the index date if the patients did not have all-cause mortality during the 5 years after stroke. All-cause mortality was defined as any record of mortality or discharge against medical advice of first-stroke hospitalization without any subsequent medical record after the discharge in the patient’s longitudinal data.

### Statistical analysis

Statistical analysis was conducted using SAS Studio 3.4. The number of mortality cases divided by the number of follow-up person-years was defined as the mortality rate. Hazard ratio (HR) was the ratio of hazard rates for mortality between two levels in a variable. The risk of mortality associated with various potential factors was determined using Cox proportional hazards regression model. *P* value of < 0.05 was considered to be statistically significant.

## Results

Table 1 shows the characteristics of selected potential factors in the collected 3,956 stroke patients.

**Table 1:** Characteristics of patients with stroke (n = 3,956).

Characteristics	Number (%)
Body mass index (kg/m^2^)	
<18.5	163 (4.1%)
18.5–24.9	2072 (52.4%)
≥25	1721 (43.5%)
GCS score	
15	2848 (72.0%)
<15	1108 (28.0%)
Type of stroke	
Ischemic	2873 (72.6%)
Hemorrhagic	1083 (27.4%)
SSI score	
<10	2740 (69.2%)
10–19	778 (19.7%)
≥20	438 (11.1%)
CCI score	
0	2510 (63.5%)
1–2	1315 (33.2%)
≥3	131 (3.3%)
Rehabilitation during first-time hospitalization	
Yes	2142 (54.1%)
No	1814 (45.9%)
Body temperature (°C)	
<35.7	164 (4.1%)
35.7–37.3	3461 (87.5%)
>37.3	331 (8.4%)
Systolic blood pressure (mmHg)	
<120	359 (9.1%)
120–139	875 (22.1%)
≥140	2722 (68.8%)
Diastolic blood pressure (mmHg)	
<80	1498 (37.9%)
80–89	997 (25.2%)
≥90	1461 (36.9%)
Blood sugar (mmol/L)	
<3.89	21 (0.5%)
3.89–7.77	2743 (69.4%)
>7.77	1192 (30.1%)
Hemoglobin (g/L)	
<120	576 (14.6%)
120–175	3303 (83.5%)
>175	77 (1.9%)
Mean corpuscular volume (fL)	
<80	305 (7.7%)
80–100	3537 (89.4%)
>100	114 (2.9%)
Red cell distribution width	
<0.115	10 (0.3%)
0.115–0.145	3407 (86.1%)
>0.145	539 (13.6%)
Red blood cell count (10^12^/L)	
<4.0	523 (13.2%)
4.0–5.9	3293 (83.3%)
>5.9	140 (3.5%)
White blood cell count (10^9^/L)	
<3.5	26 (0.6%)
3.5–11.0	3156 (79.8%)
>11.0	774 (19.6%)
Serum creatinine (μmol/L)	
<38.90	14 (0.3%)
38.90–112.27	3183 (80.5%)
>112.27	759 (19.2%)
Serum sodium (mmol/L)	
<134	203 (5.1%)
134–148	3737 (94.5%)
>148	16 (0.4%)
Serum potassium (mmol/L)	
<3.6	1184 (29.9%)
3.6–5.0	2710 (68.5%)
>5.0	62 (1.6%)
Age (years)	
<45	445 (11.2%)
45–64	1707 (43.2%)
≥65	1804 (45.6%)
Sex	
Men	2442 (61.7%)
Women	1514 (38.3%)
Insured amount per month	
<USD 700	3255 (82.3%)
≥USD 700	701 (17.7%)
Urbanization residency level	
Urban	1946 (49.2%)
Suburban	1436 (36.3%)
Rural	574 (14.5%)
Hospital accreditation level	
Medical center	3399 (85.9%)
Regional hospital	557 (14.1%)

SD = standard deviation; GCS = Glasgow coma scale; SSI = Stroke Severity Index; CCI = Charlson–Deyo Comorbidity Index; USD = United States dollar.

Table 2 illustrates the HR for mortality with regard to selected potential factors. An increased risk of mortality in patients with stroke was observed with the following: BMI <18.5 kg/m^2^ (adjusted HR, 1.33; 95% confidence interval [CI], 1.02–1.73; *P* < 0.05), GCS score <15 (adjusted HR, 1.43; 95% CI, 1.18–1.72; *P* < 0.01), SSI score 10–19 (adjusted HR, 2.85; 95% CI, 2.32–3.49; *P* < 0.01), SSI score ≥20 (adjusted HR, 3.92; 95% CI, 3.05–5.03; *P* < 0.01), CCI score 1–2 (adjusted HR, 1.65; 95% CI, 1.39–1.96; *P* < 0.01), CCI score ≥3 (adjusted HR, 4.21; 95% CI, 3.26–5.44; *P* < 0.01), no rehabilitation during first-stroke hospitalization (adjusted HR, 1.86; 95% CI, 1.60–2.16; *P* < 0.01), blood sugar <3.89 mmol/L (70 mg/dL) (adjusted HR, 2.57; 95% CI, 1.35–4.89; *P* < 0.01), hemoglobin <120 g/L (adjusted HR, 1.34; 95% CI, 1.03–1.73; *P* < 0.05), RDW >0.145 (adjusted HR, 1.71; 95% CI, 1.40–2.08; *P* < 0.01), serum creatinine >112.27 μmol/L (adjusted HR, 1.76; 95% CI, 1.48–2.09; *P* < 0.01), serum sodium <134 mmol/L (adjusted HR, 1.73; 95% CI, 1.36–2.19; *P* < 0.01), and age ≥65 years (adjusted HR, 2.25; 95% CI, 1.88–2.69; *P* < 0.01). However, a decreased risk of mortality was observed with BMI ≥25 kg/m^2^ (adjusted HR, 0.84; 95% CI, 0.71–0.98; *P* < 0.05) and hemorrhagic stroke (adjusted HR, 0.73; 95% CI, 0.60–0.89; *P* < 0.01).

**Table 2:** Hazard ratio of mortality in relation to selected potential factors.

Potential factors	Death rate (per 1000 person-years)	Unadjusted		Adjusted*	
		Hazard ratio	95% CI	Hazard ratio	95% CI
Body mass index (kg/m^2^)					
<18.5	111.5	2.33^‡^	1.80–3.01	1.33^†^	1.02–1.73
18.5–24.9	47.8	1.00		1.00	
≥25	31.0	0.65^‡^	0.56–0.76	0.84^†^	0.71–0.98
GCS score					
15	29.1	1.00		1.00	
<15	82.9	2.86^‡^	2.48–3.30	1.43^‡^	1.18–1.72
Type of stroke					
Ischemic	39.8	1.00		1.00	
Hemorrhagic	49.8	1.26^‡^	1.08–1.47	0.73^‡^	0.60–0.89
SSI score					
<10	25.6	1.00		1.00	
10–19	79.6	3.12^‡^	2.65–3.68	2.85^‡^	2.32–3.49
≥20	105.9	4.18^‡^	3.47–5.02	3.92^‡^	3.05–5.03
CCI score					
0	28.6	1.00		1.00	
1–2	56.3	1.96^‡^	1.69–2.29	1.65^‡^	1.39–1.96
≥3	281.2	9.85^‡^	7.85–12.37	4.21^‡^	3.26–5.44
Rehabilitation during first-time hospitalization					
Yes	37.2	1.00		1.00	
No	49.1	1.33^‡^	1.16–1.54	1.86^‡^	1.60–2.16
Body temperature (°C)					
<35.7	66.0	1.68^‡^	1.24–2.27	0.98	0.71–1.34
35.7–37.3	39.7	1.00		1.00	
>37.3	61.8	1.57^‡^	1.25–1.96	1.14	0.90–1.44
Systolic blood pressure (mmHg)					
<120	53.7	1.46^‡^	1.12–1.92	1.15	0.87–1.53
120–139	36.8	1.00		1.00	
≥140	42.9	1.17	0.97–1.40	1.12	0.92–1.37
Diastolic blood pressure (mmHg)					
<80	47.3	1.12	0.94–1.34	0.97	0.80–1.18
80–89	42.0	1.00		1.00	
≥90	37.9	0.90	0.75–1.09	0.93	0.76–1.14
Blood sugar (mmol/L)					
<3.89	125.0	3.67^‡^	1.96–6.87	2.57^‡^	1.35–4.89
3.89–7.77	34.0	1.00		1.00	
>7.77	62.4	1.85^‡^	1.60–2.13	1.11	0.94–1.31
Hemoglobin (g/L)					
<120	112.2	3.39^‡^	2.91–3.95	1.34^†^	1.03–1.73
120–175	33.0	1.00		1.00	
>175	19.0	0.57	0.27–1.21	0.62	0.28–1.37
Mean corpuscular volume (fL)					
<80	39.0	0.93	0.71–1.23	0.83	0.59–1.18
80–100	42.0	1.00		1.00	
>100	67.4	1.61^‡^	1.13–2.29	1.19	0.81–1.76
Red cell distribution width					
<0.115	69.8	1.93	0.62–6.02	1.23	0.39–3.92
0.115–0.145	36.1	1.00		1.00	
>0.145	88.2	2.43^‡^	2.06–2.87	1.71^‡^	1.40–2.08
Red blood cell count (10^12^/L)					
<4.0	117.9	3.47^‡^	2.97–4.06	1.25	0.97–1.61
4.0–5.9	33.8	1.00		1.00	
>5.9	18.0	0.53^†^	0.30–0.95	0.62	0.33–1.19
White blood cell count (10^9^/L)					
<3.5	131.9	3.44^‡^	1.94–6.10	1.61	0.89–2.89
3.5–11.0	37.8	1.00		1.00	
>11.0	60.3	1.61^‡^	1.37–1.90	1.09	0.90–1.31
Serum creatinine (μmol/L)					
<38.90	49.2	1.56	0.50–4.86	1.79	0.56–5.73
38.90–112.27	32.2	1.00		1.00	
>112.27	91.0	2.82^‡^	2.43–3.27	1.76^‡^	1.48–2.09
Serum sodium (mmol/L)					
<134	119.8	3.06^‡^	2.45–3.82	1.73^‡^	1.36–2.19
134–148	39.0	1.00		1.00	
>148	59.7	1.57	0.59–4.19	1.34	0.49–3.65
Serum potassium (mmol/L)					
<3.6	46.3	1.18^†^	1.01–1.38	1.16	0.99–1.37
3.6–5.0	39.3	1.00		1.00	
>5.0	125.6	3.16^‡^	2.16–4.63	1.05	0.69–1.58
Age (years)					
<45	21.3	0.80	0.58–1.11	0.82	0.58–1.14
45–64	26.7	1.00		1.00	
≥65	64.1	2.38^‡^	2.03–2.80	2.25^‡^	1.88–2.69
Sex					
Men	40.2	1.00		1.00	
Women	46.1	1.14	0.99–1.32	0.85	0.73–1.00
Insured amount per month					
<USD 700	45.6	1.00		1.00	
≥USD 700	28.6	0.63^‡^	0.51–0.78	1.10	0.87–1.39
Urbanization residency level					
Urban	38.5	1.00		1.00	
Suburban	43.3	1.12	0.96–1.32	0.99	0.84–1.16
Rural	54.2	1.40^‡^	1.15–1.71	1.12	0.90–1.39
Hospital accreditation level					
Medical center	41.1	1.00		1.00	
Regional hospital	50.9	1.24^†^	1.02–1.50	1.05	0.85–1.30

CI = confidence interval; GCS = Glasgow coma scale; SSI = Stroke Severity Index; CCI, Charlson–Deyo Comorbidity Index; USD = United States dollar.

*Adjusted for age, sex, body mass index, GCS score, body temperature, systolic blood pressure, diastolic blood pressure, type of stroke, SSI score, CCI score, rehabilitation during first-time hospitalization, blood sugar, red blood cell count, mean corpuscular volume, red cell distribution width, hemoglobin, white blood cell count, serum creatinine, serum sodium, serum potassium, insured amount, urbanization residency level, and hospital accreditation level.

^†^P < 0.05. ^‡^P < 0.01.

## Discussion

This is the first study using comprehensive information regarding first-stroke hospitalization by connecting hospital databases with NHIRD to identify the most important factors associated with 5-year mortality in patients with stroke. The main findings of this study are that baseline data such as higher RDW, lower hemoglobin levels, lower blood sugar levels, higher serum creatinine levels, lower serum sodium levels, lower BMI, lower GCS score, greater stroke severity, more comorbidities, no rehabilitation during first-stroke hospitalization, and older age at onset can be risk factors for mortality within five years following first stroke. Higher BMI and hemorrhagic stroke can decrease 5-year mortality in patients with first stroke.

Few studies have previously reported that red blood cell parameters can affect mortality after stroke. Ani & Ovbiagele^17^ showed that a higher RDW level (>13.90% vs. ≤12.75%) among patients with stroke independently predicted subsequent all-cause mortality (HR = 2.0) in a mean follow-up period of 60 months. An increase in RDW was often considered to occur because of impaired red cell generation, which indicated a patient’s suboptimal health condition and limited capability of disease recovery^28^. In a meta-analysis by Barlas *et al*., anemia at admission was found to be associated with increased mortality for up to 1 year in patients with stroke^29^. One study showed that anemia can impair cerebrovascular autoregulation, resulting in inconsistent blood perfusion and increased brain damage^30^. Our present comprehensive large-sample-size study surveying complete red blood cell parameters and other possible factors demonstrated that higher RDW and lower hemoglobin levels were factors influencing long-term mortality in patients with stroke. We believe that this finding can be an important clinical reference when caring for patients with stroke.

One animal study illustrated that the occurrence of hypoglycemia in a cat model caused larger brain infarcts and an increased mortality rate^31^. Preclinical research implied that hypoglycemia possibly increased the risk of stroke in diabetic patients^32^. Overuse of medication to decrease blood sugar can result in hypoglycemia in patients with diabetes. Our present study demonstrated that the baseline data of blood sugar <3.89 mmol/L (70 mg/dL) after admission was a factor influencing 5-year mortality in patients with stroke. This finding was consistent with a meta-analysis suggesting that hypoglycemia was associated with increased mortality in critically ill patients^33^.

Previous literature has discussed that impaired renal function affects long-term mortality in patients with stroke. Estimated glomerular filtration rate <60 mL/min/1.73 m^2^, calculated with a formula using serum creatinine levels in the 3 days after acute stroke, was a significant predictor of 3-year mortality (HR = 1.67)^34^. Although the serum creatinine level can be affected by other factors and may not precisely reflect renal function, it currently remains as the first-line routine evaluation of renal function in patients with acute stroke.

Huanh *et al*. showed that hyponatremia (serum sodium ≤134 mmol/L) during the 3 days post-acute-stroke was a significant predictor of 3-year mortality in patients with stroke after adjusting for related variables (HR = 2.23)^35^. Soiza *et al*. illustrated that severe hyponatremia (serum sodium <125 mmol/L) was a predictor of 1-year mortality in patients with stroke^16^. However, hypernatremia (serum sodium ≥145 mmol/L) did not influence mortality in patients with stroke in the model of adjusted hematological and biochemistry data in the study by Soiza *et al*.^16^ Therefore, hyponatremia but not hypernatremia was a factor influencing long-term mortality in patients with stroke, and the findings of our present study are consistent with the abovementioned studies. However, the manner in which hyponatremia in stroke affects the long-term mortality rate is yet unknown. One hypothesis was that hyponatremia frequently accompanies pulmonary diseases, which can result in an increased mortality rate^36^. However, evidence to support this hypothesis is insufficient to date.

This study showed that stroke patients with a lower BMI had higher 5-year mortality, whereas those with a higher BMI had lower 5-year mortality; this result is consistent with that of a 4-year follow-up research by Ryu *et al*.^37^ who revealed that the all-cause mortality rate was inversely associated with BMI in patients with ischemic stroke. Compared with BMI 18.5–22.9 kg/m^2^, Ryu *et al*.^37^ found that age- and sex-adjusted HR was 2.54 for BMI <18.5 kg/m^2^ and 0.60 for BMI ≥25 kg/m^2^. Baseline low BMI may reflect low muscle mass^38^ and inadequate nutrition^39^, which are detrimental factors for patients with stroke.

The impairment of consciousness after stroke reportedly affects long-term mortality in patients with stroke. Vemmos *et al*.^40^ illustrated that lower GCS was one of the most powerful predictors of 1-year mortality in patients with stroke. Our result was similar to that reported by Vemmos *et al*. Furthermore, previous studies have demonstrated that stroke severity can affect long-term survival rate in patients with stroke. Sarbazi *et al*. showed that stroke severity measured by the National Institutes of Health Stroke Scale was the most important factor for predicting 6-month mortality in patients with first stroke^41^. Using the Scandinavian Stroke Scale to assess stroke severity, Mogensen *et al*. illustrated that 10-year mortality in patients with stroke was associated with greater stroke severity^42^. Although different tools were used to measure stroke severity, our present study showed that higher long-term stroke mortality was associated with greater stroke severity.

The effect of comorbidity on mortality can continue for a long period after stroke, and this finding was consistent with that in previous studies. Corraini *et al*. showed that a higher CCI score resulted in higher post-stroke mortality in patients with stroke during the first year of follow-up^43^. Schmidt *et al*. illustrated that, compared with patients with a CCI score of 0, the 5-year mortality rate ratio increased 1.46-fold, 1.69-fold, and 2.47-fold in those with a CCI score of 1, 2, and 3, respectively^44^. Our present study showed that compared with patients with a CCI score of 0, patients with more comorbidities (higher CCI scores) were markedly at a higher risk of 5-year mortality after stroke. Accordingly, our findings corroborate the results of the study by Schmidt *et al*.

Our present study showed that no rehabilitation during first-stroke hospitalization can increase mortality rates within 5 years after stroke. The effect of early rehabilitation on mortality after stroke has been discussed in previous literature. One population-based study showed that rehabilitation in the first 3 months after stroke can decrease the 10-year mortality rate in patients with stroke^45^. Another population-based study illustrated that first-stroke survivors who received rehabilitation during first-stroke hospitalization, either transferring to rehabilitation ward or not, had a decreased 5-year mortality rate than patients who did not receive any rehabilitation during first-stroke hospitalization^4^. Early rehabilitation, particularly during first-stroke hospitalization, may result in long-term benefits by decreasing the mortality rate.

Andersen *et al*. reported that patients with hemorrhagic stroke had a considerably higher crude 3-month mortality rate than patients with ischemic stroke^46^. However, the results of the study by Andersen *et al*. were not adjusted for other factors. Moreover, our study also showed that hemorrhagic stroke was associated with a higher crude mortality rate than ischemic stroke. Because most hemorrhagic stroke cases had greater stroke severity than ischemic stroke cases at stroke onset, hemorrhagic stroke was likely to have a higher mortality rate after stroke. However, hemorrhagic stroke had a lower risk of mortality than ischemic stroke after adjustment of baseline stroke severity and other factors.

Our study has some limitations because of its retrospective database analysis design. Some patients had missing data for baseline markers, and these patients were excluded from the study; however, this may result in a bias. Second, all possible factors were not included in the analysis. Some clinical information, such as smoking status, was not recorded and was not included in the analysis. Additionally, the recombinant tissue-type plasminogen activator (rt-PA) therapy, one of the concomitant treatments, was not included for analysis because rt-PA is specific to patients with ischemic stroke of which very few patients were treated with rt-PA during our data collection period (2004–2008). Third, the diagnosis of stroke was based on ICD-9-CM codes, which were not accurate enough to allow the classification of patients into different types or sides of stroke. Despite the limitations, the article clearly identifies the most important factors affecting long-term mortality in patients with stroke based on their clinical data, laboratory data, demographic/socioeconomic status, and hospital accreditation level.

## Conclusions

This comprehensive study demonstrated that following baseline data present factors influencing the five-year mortality rate in patients with first stroke: RDW >0.145, hemoglobin <120 g/L, blood sugar <3.89 mmol/L (70 mg/dL), serum creatinine >112.27 μmol/L, serum sodium <134 mmol/L, BMI <18.5 kg/m^2^, BMI ≥25 kg/m^2^, GCS score <15, age ≥65 years with SSI score ≥10, CCI score ≥1, and no rehabilitation during first-stroke hospitalization. Patients with stroke with such conditions at baseline may require more intensive care and risk-factor control to decrease their long-term mortality.

## Data Availability

The data generated during the current study are available from the corresponding author on reasonable request.
